# ArthropodaCyc: a CycADS powered collection of BioCyc databases to analyse and compare metabolism of arthropods

**DOI:** 10.1093/database/baw081

**Published:** 2016-05-30

**Authors:** Patrice Baa-Puyoulet, Nicolas Parisot, Gérard Febvay, Jaime Huerta-Cepas, Augusto F. Vellozo, Toni Gabaldón, Federica Calevro, Hubert Charles, Stefano Colella

**Affiliations:** ^1^ Univ Lyon, INSA-Lyon, INRA, BF2I, UMR0203, F-69621, Villeurbanne, France; ^2^ Centre for Genomic Regulation (CRG), the Barcelona Institute of Science and Technology, Dr. Aiguader 88, Barcelona 08003, Spain; ^3^ Univ Lyon, Univ Lyon1, CNRS, LBBE, UMR5558, F-69622, Villeurbanne, France; ^4^ Universitat Pompeu Fabra (UPF), Barcelona 08003, Spain; ^5^Institució Catalana de Recerca i Estudis Avançats (ICREA), Pg. Lluís Companys 23, Barcelona 08010, Spain

## Abstract

Arthropods interact with humans at different levels with highly beneficial roles (e.g. as pollinators), as well as with a negative impact for example as vectors of human or animal diseases, or as agricultural pests. Several arthropod genomes are available at present and many others will be sequenced in the near future in the context of the i5K initiative, offering opportunities for reconstructing, modelling and comparing their metabolic networks. In-depth analysis of these genomic data through metabolism reconstruction is expected to contribute to a better understanding of the biology of arthropods, thereby allowing the development of new strategies to control harmful species. In this context, we present here ArthropodaCyc, a dedicated BioCyc collection of databases using the Cyc annotation database system (CycADS), allowing researchers to perform reliable metabolism comparisons of fully sequenced arthropods genomes. Since the annotation quality is a key factor when performing such global genome comparisons, all proteins from the genomes included in the ArthropodaCyc database were re-annotated using several annotation tools and orthology information. All functional/domain annotation results and their sources were integrated in the databases for user access. Currently, ArthropodaCyc offers a centralized repository of metabolic pathways, protein sequence domains, Gene Ontology annotations as well as evolutionary information for 28 arthropod species. Such database collection allows metabolism analysis both with integrated tools and through extraction of data in formats suitable for systems biology studies.

**Database URL:** http://arthropodacyc.cycadsys.org/

## Introduction

More than 10 years have passed since the publication of the initial sequencing and analysis of the human genome (1, 2), which has had a great impact on the way we investigate biological processes, notably with the development of novel technologies enabling comprehensive genomic analyses (3). The genomes of several other organisms were sequenced before and after the human genome, starting with the animal models: *Drosophila melanogaster* (4), *Mus musculus* (5) and *Rattus norvegicus* (6). These data are driving the development of genomics-based research approaches to study the biology of many living organisms beyond humans and established model organisms. Recently, a large community of researchers have launched the Genome 10K project to obtain the full genome sequences of 10 000 vertebrate species (7). A similar initiative has been launched by the Arthropod Genomics consortium: the i5K initiative, which aims at sequencing the genomes of 5000 arthropod species (http://arthropodgenomes.org/wiki/i5K) (8, 9).

The availability of the full genome sequence of an organism allows researchers to have a complete view of its metabolism. The BioCyc collection of Pathway/Genome DataBases (PGDBs) (10) constitutes a key resource for studying the metabolism of multiple organisms as it enables comparative studies. The first database of the collection, EcoCyc (11), is at present a comprehensive resource to study *Escherichia coli* biology (12). The quality of these databases is strongly linked to the annotation used to generate them and, in the most recent release (June 24, 2015—version 19.1), only seven databases are intensively manually curated and frequently updated (BioCyc Tier 1 PGDBs): EcoCyc (12), MetaCyc (13), HumanCyc (14), AraCyc (15), YeastCyc (16), LeishCyc (17) and TrypanoCyc (18). Such expert driven annotation is only possible for large communities of scientists working on the same model and, consequently, the majority of the 5711 BioCyc PGDBs available in this release are computationally derived: 39 are subject to moderate manual curation (BioCyc Tier 2 PGDBs) and 5455 to no manual curation at all (BioCyc Tier 3 PGDBs) (see http://biocyc.org/biocyc-pgdb-list.shtml for an updated listing). The upcoming deluge of fully sequenced genomes, driven by NGS technology, demands the development of a novel genomic infrastructure (19). To contribute to the need of standardized automated annotation, we developed a Cyc Annotation Database System (CycADS) (20). CycADS was successfully used to generate AcypiCyc (http://acypicyc.cycadsys.org), a database dedicated to the pea aphid *Acyrthosiphon pisum* metabolism that was developed during the annotation phase of the genome of this insect (21). As many other arthropod genomes are available, and many more will be in the future in the context of the i5K initiative, we decided to develop a collection of BioCyc Tier 3 pathway/genome databases for arthropods using the uniform and enriched automated functional annotations provided by the CycADS system.

## Implementation

### CycADS annotation management system

**Figure 1.**
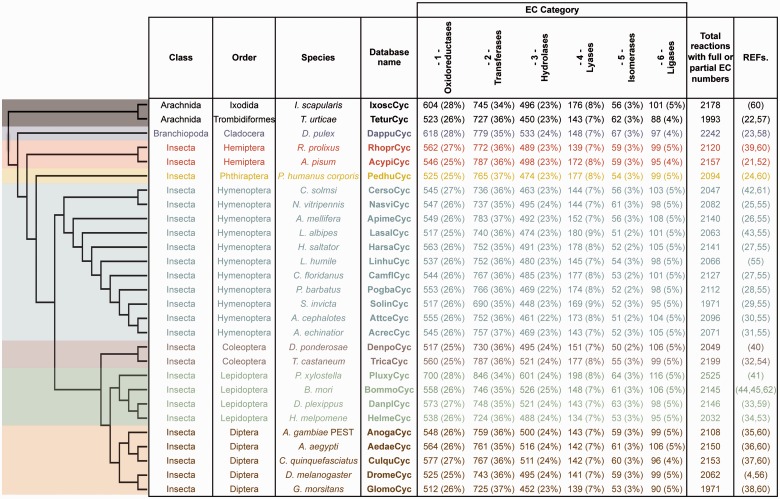
ArthropodaCyc databases list and summary. This table shows the distribution of reactions in the Cyc databases across the six top-level categories identified by the Enzyme Commission (E.C.). Included in this table are all reactions in each database which have been assigned either full or partial E.C. numbers, and for which an enzyme has been identified (these statistics do not include pathway holes). Phylogenetic relationships between species are displayed using a cladogram based on available data (46–50).

ArthropodaCyc is a collection of BioCyc PGDBs that contains, at the time of writing (March 2016), the metabolic network of 28 arthropods with sequenced genomes (4, 21–45), including 25 insects, two arachnids and a branchiopod [Figure 1, note that phylogenetic relationships between species are displayed using a cladogram based on available data (46–50)]. All databases in ArthropodaCyc were generated using CycADS (20), an annotation management system programmed in Java (Model-View-Controller structure) and SQL that was originally developed for the annotation of the pea aphid genome (21). CycADS facilitates the collection and management of information obtained from both genomic data and different protein annotation methods in a SQL database. A pipeline to filter for bacterial contaminants was developed and integrated in the protein functional annotation system, which involves multiple methods (see below for a detailed description). All data collected in CycADS were then extracted and formatted to generate, for each organism, an *ad hoc* input file (a BioCyc ‘Path-o-logic file’ format) used by the Pathway Tools software (51) to produce BioCyc-like enriched metabolic database (20) (Supplementary Figure S1). Genomes are included in ArthropodaCyc if they fulfil the following criteria: (i) the genome sequence is published and (ii) the sequence data can be downloaded in appropriate formats (comprehensive GFF or Genbank file with compatible gene/mRNA/protein features). Each organism database summary page contains updated information relative to the genome data release used to generate it.

### Filtering possible bacterial contaminations

Organisms across all kingdoms of life are associated with microbial partners, with interactions ranging from parasitism to mutualism. Arthropods are no exception and can harbour microorganisms at their external surface, or internally as endosymbionts, gut microbiota, parasites or pathogens. Despite the use of specific DNA extraction protocols, massive sequencing of arthropod genomes may generate sequences contaminated by prokaryotic DNA. Since the ArthropodaCyc databases aims at collecting high-quality functional annotations, we decided to implement a pipeline for the detection of putative contaminant bacterial sequences, to be used before the reconstruction step of the arthropod metabolic pathways.

First, genomic sequences, annotation files (GFF/GBK) and protein sets of each arthropod genome project were retrieved from public repositories (52–62). Genomic sequences smaller than 50 kbp were compared to the NCBI’s RefSeq prokaryotic genome sequences database (63) using BLASTN (64). Contaminant genomic sequences were identified using a 90% identity threshold over at least 90% of the query length. A BLASTP against the NCBI RefSeq prokaryotic protein sequences database was thus performed to check for bacterial contaminations within the remaining proteins. Protein sequences were filtered based on BLAST results using three different criteria: (i) at least 90% amino acid identity over at least 90% of the query length, (ii) at least 90% amino acid identity over at least 50% of both the query and the hit lengths or (iii) at least 95% identity over a sliding window of 100 amino acids. To reduce the risk of removing arthropod sequences (false-positives), we included a last step performing a BLASTP (≥80% amino acid identity over at least 80% of the query length) of the putative contaminant protein list against an invertebrate subset of the reference UniProtKB/Swiss-Prot protein sequence database (65). Proteins with positive hits were reintegrated in the annotation process. Lists of putative bacterial contaminants detected within the 28 arthropod genomes of ArthropodaCyc are provided in Supplementary Table S1. The proteins identified as putative bacterial contaminants were flagged upon extraction and they were not used in the Pathway Tools reactions inference (see below). However, they still appear in the database with a gene/protein page and the information about their status of contaminants.

### The functional annotation pipeline

We used multiple methods to perform a functional annotation: the online KAAS-KEGG annotation pipeline (66) and the PRIAM (67), Blast2GO (68, 69) and InterProScan (70) pipelines with a local installation for faster data generation (summary of results in Supplementary Table S2). These methods generated functional information (EC number, KEGG Orthology and Gene Ontology) related to the protein sequences and all annotations were collected in the database using flexible annotation loaders (annotation collector module) available in CycADS. Default parameters were used for software configurations and the BLAST alignments (prior to the Blast2GO analysis) were performed against the reference UniProtKB/Swiss-Prot protein sequences database (65). All annotation data were extracted from the CycADS SQL database for each analysed genome and collected in a Pathologic file that was used in Pathway Tools (51) to generate the corresponding BioCyc PGDB.

For several arthropods, a genome wide phylogenetic analysis performed using the Phylome pipeline and collected in PhylomeDB (71) was available. In those cases, Gene Ontology annotations were transferred using orthology relationships to *Drosophila melanogaster* and integrated in the ArthropodaCyc databases using CycADS as previously described (20). Furthermore, for the arthropods with a Phylome analysis, the orthology predictions generated in MetaPhOrs (June 2015 release) (72) using a combination of phylogenetic information derived from different databases were included into the corresponding BioCyc databases using the orthology functionality of Pathway Tools.

**Figure 2.**
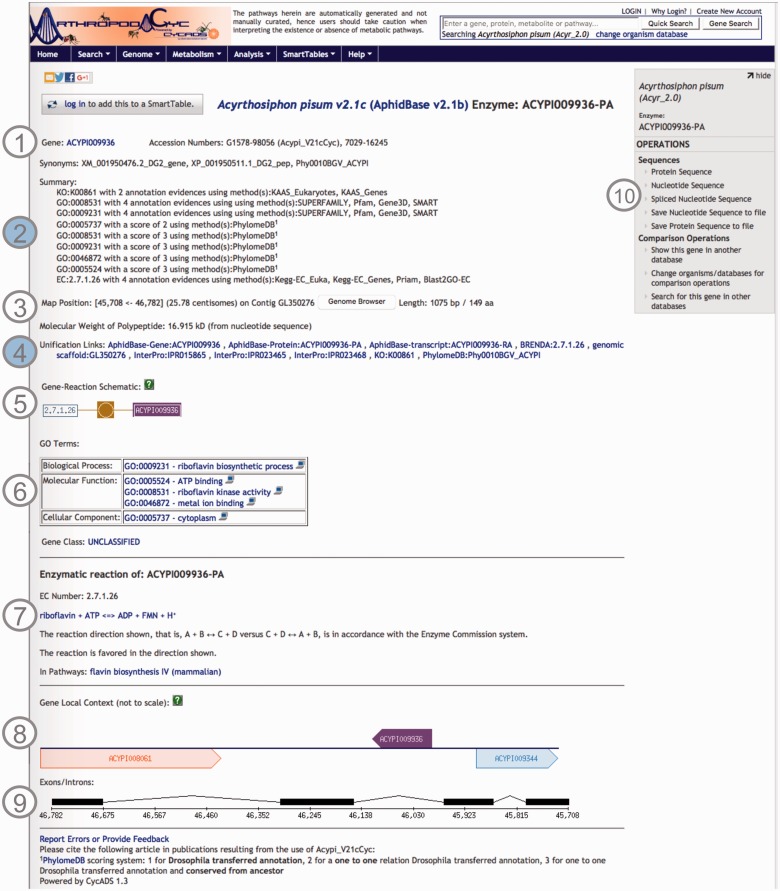
Screenshot of an ArthropodaCyc enzyme page. The page provides several information such as: (1) gene name, accession numbers and synonym names; (2) a summary of metabolism annotation evidences from KAAS-KEGG, PRIAM, InterProScan, PhylomeDB and BLAST2GO; (3) genome position with an additional link to the corresponding genome browser, and information on gene and protein length and polypeptide molecular weight; (4) external cross-links to specific genomic databases, enzyme annotation and InterProScan domains information and to phylogeny in PhylomeDB; (5) schematics representing the reaction(s) carried out by the enzyme; (6) Gene ontology terms associated with the enzyme functions; (7) additional information on the reaction(s) carried out by the enzyme, including the pathway(s) (if any) where this reaction may occur; (8) gene local context, including neighbouring genes; (9) gene structure in terms of (added) exons/introns organization and (10) an “Operations box” offering several options for comparative analyses. Filled circles, (2) and (4), represent ArthropodaCyc specific features.

Using CycADS, enriched gene records were automatically generated in the Pathologic file format imported by Pathway Tools. Each Pathologic file record contained the gene and the gene product names, synonyms, sequence structural information, as well as the annotations including Enzyme evidences (E.C. numbers), KEGG Orthology, Gene Ontology and MetaPhOrs orthology that were integrated in the databases. In the note section for each gene/protein page, the information relative to the annotation results are recorded to allow the researchers to evaluate the confidence for each putative function assigned to a protein (20). Useful external cross-links, such as to NCBI’s RefSeq or genomic databases of arthropod communities (i.e. AphidBase, VectorBase and Hymenoptera Genome Database), are also integrated thanks to the CycADS pipeline. Moreover, as InterProScan (70) analysis identifies functional domains, we included links to the InterPro external database identifiers when appropriate (see Figure 2 example page).

It is important to underline that, as data formats in genomics can be very disparate depending on the source file format used (i.e. GFF, Gbk), data on each arthropod genome were manually checked to ensure that a unique identifier for genes and products was present and that clear relations could be established among the different features. The flexible CycADS parsers were parameterized using its detailed configuration file (20). Finally, all automatic tasks of Pathway Tools consistency checker were run before databases saving and publishing. With the perspective to make available an up-to-date database representative of the fast evolving field of arthropod genomics, ArthropodaCyc will be updated annually.

## Discussion on features and usage

The ArthropodaCyc collection of enriched BioCyc databases for arthropods whose genome has been fully sequenced and assembled is a key resource for all members of the Arthropod Genomics Consortium (http://arthropodgenomes.org/wiki/Main_Page). Our collection takes advantage of CycADS (20): a powerful annotation management system allowing to manage multiple genomes and to generate a set of BioCyc database where each organism has been annotated using identical tools and automatized procedures. Furthermore, our collection is enriched by phylogeny-based orthology predictions available in PhylomeDB/MetaPhOrs (71, 72) and customized hyperlinks to organism specific genome browsers. The ArthropodaCyc collection of databases takes also full advantage of the rich BioCyc interface and tools for metabolism data analysis (73). Several analyses can be performed using the BioCyc online interface that includes advanced query tools (74), and powerful web-based genomic data viewers (75). Moreover, ArthropodaCyc offers the possibility to download the data in formats suitable for data analysis: either using other tools, such as for example Cytoscape (76) and MetExplore (77), or for use in personally developed analysis software and pipelines.

We are already using ArthropodaCyc to contribute to the analysis of the metabolism in genome annotation projects currently ongoing on different insect species: the green peach aphid *Myzus persicae* [manuscript in preparation], the milkweed bug *Oncopeltus fasciatus* [manuscript in preparation](78–80) and the rice weevil *Sitophilus oryzae*. Beyond the single organism analysis, the BioCyc interface provides the user with tools for comparative analyses (51) to identify interesting features of a given organism metabolism that could shed light on its biology. The interest in comparative analyses will greatly grow as more arthropods are sequenced.

**Figure 3.**
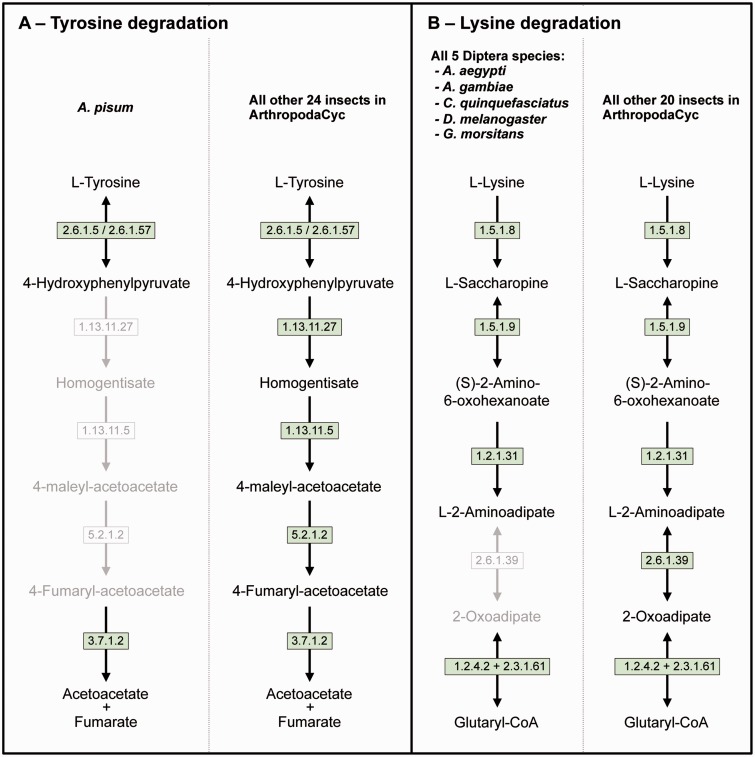
Two examples of insect pathway differences identified using ArthropodaCyc. (A) Pathway of tyrosine degradation, comparison between *A. pisum* and the other insects of ArthropodaCyc; (B) Pathway of lysine degradation, comparison between the five species of Diptera and the other insects of ArthropodaCyc. In each pathway, green coloured enzymes are present, while grey enzymes and reactions are absent. Enzymes: 1.13.11.5 = homogentisate 1,2-dioxygenase; 1.13.11.27 = 4-hydroxyphenylpyruvate dioxygenase; 1.2.1.31 = L-aminoadipate-semialdehyde dehydrogenase; 1.2.4.2 = oxoglutarate dehydrogenase (succinyl-transferring); 1.5.1.8 = saccharopine dehydrogenase (NADP+, L-lysine-forming); 1.5.1.9 = saccharopine dehydrogenase (NAD+, L-glutamate-forming); 2.3.1.61 = dihydrolipoyllysine-residue succinyltransferase; 2.6.1.5 = tyrosine transaminase; 2.6.1.39 = 2-aminoadipate transaminase; 2.6.1.57 = aromatic-amino-acid transaminase; 3.7.1.2 = fumarylacetoacetase; 5.2.1.2 = maleylacetoacetate isomerase.

Even if a full comparative analysis in arthropods is beyond the purpose of this article, we provide here a few examples of its usage. ArthropodaCyc can be used for the identification of enzymes and/or pathways unique to a given organism or group of organisms. As an example, we used the present version of the ArthropodaCyc database to verify the lack of the tyrosine degradation pathway that we had originally described in the pea aphid genome by using the AcypiCyc database (20, 21) and comparing this genome to the few insect genomes available at that time. We could confirm that *A. pisum* is the only insect lacking this pathway among the 25 available in the database at present (Figure 3A). We interpreted this loss as an explanation of the pea aphid lifestyle and we linked it to the high demand for tyrosine by pea aphids in connection with their unbalanced plant phloem sap diet. Even though the nutrition of these insects is complemented by their primary symbiont, *Buchnera aphidicola*, this bacterium provides only precursors for tyrosine biosynthesis. The enzymes catalysing the last two steps of tyrosine synthesis are in fact encoded in the aphid genome. In particular, multiple genes coding the aspartate transaminase (E.C. 2.6.1.1), the enzyme involved in the synthesis of phenylalanine from phenylpyruvate are present in the pea aphid genome with one of them (*ACYPI004243*) specifically regulated during embryo development in aphids reproducing by parthenogenesis (81). We thus checked whether this aspartate transaminase gene expansion was also present in the genomes of the other 27 arthropods present in the ArthropodaCyc database. Remarkably, only six out of the other 27 arthropods show a gene expansion comparable to the pea aphid (5 or 4 genes), and all the other 21 arthropods present only 2 or 3 genes encoding for this enzyme (Supplementary Table S3). Even if further phylogenetic analyses would be needed to better understand the origin of the differences in this enzyme-coding gene, this is an example of another possible use of ArthropodaCyc to easily explore the number of genes and their structures for specific enzymes (an interesting genome variation beyond the relatively rare presence/absence case of enzymatic reactions).

As another example application, we also decided to explore the database to search for pathways that would be characteristic of a specific group of insects and we identified the 2-aminoadipate transaminase (E.C. 2.6.1.39) in the lysine degradation pathway as uniquely missing in the genomes of the five dipteran species available in ArthropodaCyc (*A. aegypti, A. gambiae, C. quinquefasciatus, D. melanogaster* and *G. morsitans*) (Figure 3B). These examples provided here show the power of ArthropodaCyc in finding differences between specific organisms that might be linked with their biology, even though for the lysine degradation it is difficult to speculate on possible reasons for this apparent loss of a complete pathway as these five dipteran species live in multiple habitats and feed on very diversified diets.

## Conclusions

We present here ArthropodaCyc, the most comprehensive collection of BioCyc databases for arthropods, which we expect to be of great interest for a broad community of scientists. Several genomes of arthropods are being sequenced and many more will be sequenced in the future as part of the i5K initiative. The CycADS pipeline empowers both the development and the update of the PGDB in ArthropodaCyc. Our databases are an arthropod research resource that is also linked, whenever possible, to single organism community based genomic databases, thus offering to the researchers an integrated access to different sources of annotations.

## Supplementary Material

Supplementary Data

Supplementary Data
