# Supplementary figures and images for: ArthropodaCyc: a CycADS powered collection of BioCyc databases to analyse and compare metabolism of arthropods
Source: Database (Oxford). 2016 May 30;2016:baw081. doi: 10.1093/database/baw081 (PMC5630938; doi:10.1093/database/baw081)

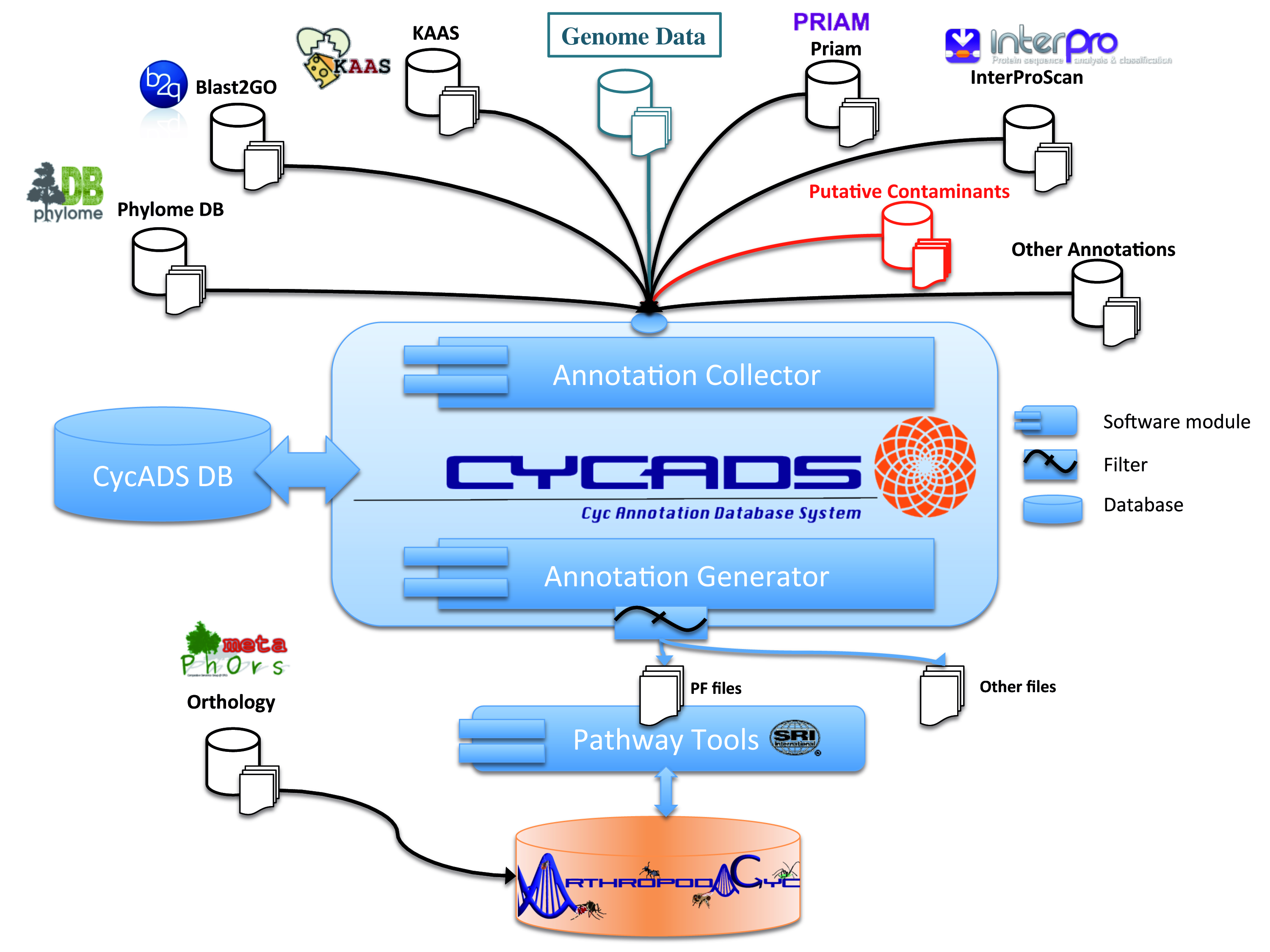

Supplement: Supplementary Data [file baw081_Supplementary_Data.zip › Supplementary_Figure_S1_fin.tif]
