# Supplementary material for: ArthropodaCyc: a CycADS powered collection of BioCyc databases to analyse and compare metabolism of arthropods
Source: Database (Oxford). 2016 May 30;2016:baw081. doi: 10.1093/database/baw081 (PMC5630938; doi:10.1093/database/baw081)
Supplement: Supplementary Data [file supp_2016_baw081_index.html]

ArthropodaCyc: a CycADS powered collection of BioCyc databases to analyse and compare metabolism of arthropods — Supplementary Data 

# ArthropodaCyc: a CycADS powered collection of BioCyc databases to analyse and compare metabolism of arthropods

## Supplementary Data

files

- Supplementary Data - zip file
